# Evolution and extinction can occur rapidly: a modeling approach

**DOI:** 10.7717/peerj.11130

**Published:** 2021-04-13

**Authors:** Vitaly A. Likhoshvai, Tamara M. Khlebodarova

**Affiliations:** 1 Department of Systems Biology, Institute of Cytology and Genetics, Siberian Branch of the Russian Academy of Sciences, Novosibirsk, Russian Federation; 2 Kurchatov Genomics Center, Institute of Cytology and Genetics, Siberian Branch of the Russian Academy of Sciences, Novosibirsk, Russian Federation

**Keywords:** Mathematical modeling, Fossil records, Punctuated evolution, Mass extinctions, Dynamic Systems, Complex dynamics, Periodicity

## Abstract

Fossil record of Earth describing the last 500 million years is characterized by evolution discontinuity as well as recurring global extinctions of some species and their replacement by new types, the causes of which are still debate. We developed a model of evolutionary self-development of a large ecosystem. This model of biota evolution based on the universal laws of living systems functioning: reproduction, dependence of reproduction efficiency and mortality on biota density, mutational variability in the process of reproduction and selection of the most adapted individuals. We have shown that global extinctions and phases of rapid growth and biodiversity stasis can be a reflection of the emergence of bistability in a self-organizing system, which is the Earth’s biota. Bistability was found to be characteristic only for ecosystems with predominant sexual reproduction. The reason for the transition from one state to another is the selection of the most adapted individuals. That is, we explain the characteristics of the Earth’s fossil record during the last 500 million years by the internal laws of Earth’s ecosystem functioning, which appeared at a certain stage of evolution as a result of the emergence of life forms with an increased adaptive diversification associated with sexual dimorphism.

## Introduction

The theory of punctuated equilibrium proposed by Stephen Jay Gould and Niles Eldredge in 1972 (Gould & Eldredge, 1977; Gould & Eldredge, 1993; Eldredge & Gould, 1997) is based on some empirical generalizations of a number of facts long noticed by paleontologists, which indicate that, in the course of evolution, long periods of stability when the main features of species remain unchanged alternate with short intervals of rapid qualitative changes. Authors of this theory and other researchers have found quite vivid examples in the fossil record confirming the existence of this pattern (Ovcharenko, 1969; Bambach, 1977; Gould & Eldredge, 1977; Gould & Eldredge, 1993; Van Valen, 1973; Sepkoski Jr, 1988; Jackson & Cheetham, 1999). Although the interpretation of some studies has been questioned (Van Bocxlaer, Damme & Feibel, 2008), in general, the existence of this pattern in the evolutionary process is not denied (Hunt, 2007; Rasskin-Gutman & Esteve-Altava, 2008; Mattila & Bokma, 2008).

It should be noted that the phenomenon of punctuated evolution is closely related to the question of evolutionary uniformitarianism. Until recently, this key question of evolutionary theory was one of the most demanding. At present, it can be considered proven that discontinuity of evolution at the paleontological level is reflected at the molecular level (Nichol, Rowe & Fitch, 1993; Pagel, Venditti & Meade, 2006; Wolf et al., 2006; Palmer et al., 2012). Moreover, it was possible to perform a quantitative assessment of the contributions of static and explosive periods to the evolutionary process. Thus, according to data (Pagel, Venditti & Meade, 2006), about 22% of the observed nucleotide differences occur during the explosive periods of speciation, while the remaining 78% gradually accumulate during the stable period of evolution. However, there is still debate about the causes of discontinuity and uneven pace of evolution (Voje, 2016; Voje, Starrfelt & Liow, 2018).

We suggested that sudden extinctions of species in different periods of the Earth’s history and their replacement by new types, known as global extinctions, are a reflection of a pattern of rapid large-scale extinction. A number of studies (Raup & Sepkoski, 1982; Raup & Sepkoski, 1984; Raup & Sepkoski Jr, 1986; Sepkoski Jr, 1989; Rohde & Muller, 2005; Lieberman & Melott, 2007) that demonstrate the periodicity of global extinctions of Earth’s biota in the last 500 million years support our idea.

Currently, at least six global extinctions have been recorded in the Earth’s fossil record. Each of them can be explained by various external reasons, for example, global climate change (MacLeod, 2003; Huey & Ward, 2005; Kaiho et al., 2016), sea level change (Hallam & Wignall, 1999; Peters, 2008), volcanic activity (Wignall et al., 2009; Courtillot & Fluteau, 2010; Rampino et al., 2017; Miller et al., 2017; Heimdal et al., 2018; Smolarek-Lach et al., 2019), meteorite impact (Alvarez et al., 1980; Alvarez et al., 1981; Schulte et al., 2010; Kaiho & Oshima, 2017), cosmic-ray flux (Lieberman & Melott, 2012), supernova explosions (Fields et al., 2020) and other causes (Huey & Ward, 2005; Archibald et al., 2010; Cermeño et al., 2017).

Here we explore the extent to which interactions that might arise from relatively simple diversity dynamics could also create patterns of mass extinction. This has been previously suggested as an idea of self-organized criticality (Bak & Paczuski, 1995; Sneppen et al., 1995; Solé & Manrubia, 1996), which assumes that interactions between different ecosystems together with spontaneous mutations can lead to large evolutionary rearrangements called “co-evolutionary avalanches”. However, Newman (1997a), Newman, (1997b) and Alroy, (2008) had arguments against this idea.

Another area that has been explored is the extent to which random, stochastic factors might have produced the pattern of biodiversity seen through time (Cornette & Lieberman, 2004; Lieberman, 2016).

There are also other concepts that suggest a certain role of predators in shaping the diversity of marine biota (Huntley & Kowalewski, 2007), although in studies (Madin et al., 2006) no such correlation was found. Our understanding of the internal causes that determine the dynamic features of the global ecosystem functioning differs from those proposed earlier (Bak & Paczuski, 1995; Sneppen et al., 1995; Solé & Manrubia, 1996; Newman, 1997a; Newman, 1997b; Sznajd-Weron & Weron, 2001; Huntley & Kowalewski, 2007). Our view was constructed using only those laws describing the processes of birth, death, reproduction, and evolution directed towards increasing the adaptability of the organism to the environmental conditions.

From this perspective, using a minimal logistic model of the global ecosystem, we managed to interpret the biodiversity stagnation observed in the Precambrian Era and the periodicity of global extinctions seen in the fossil record of life on Earth during the last 500 million years (Likhoshvai et al., 2017).

In the framework of this model, which considers same universal features of living systems, it was demonstrated that punctual changes in biodiversity are a reflection of the emergence of two different stable states (bistability) in a self-developing ecosystem. The reason for the transition from one state to another is the selection and sexual reproduction.

That is, the model predicts that sexual reproduction may be one of the reasons for the emergence of bistability in the system.

## Model of Evolutionary Self-development of a Large Ecosystem

We developed a model of an abstract ecosystem without a description of its internal structure and interacting elements. The ecosystem consists of *n* species. We assumed that the species in the ecosystem have a “self-reproducing potential”, can speciate as well as their “reproductive potential” and presumably they can go extinct to. The ecosystem is large enough so that it can be correlated with biota of the entire Earth.

The model has been developed using a modular approach where an investigated process or biological phenomenon is considered via combination of its subsystem’s models. This approach is widely used to construct diverse models of biological systems (Kurata et al., 2014; Likhoshvai et al., 2014; Kazantsev et al., 2018; Kiselev et al., 2019; Akberdin et al., 2020). The model of a large ecosystem consists of two subsystems. The first subsystem describes the self-development of a large ecosystem over time. The second subsystem describes the evolution of ecosystem parameters. Derivation of equations for self-development and evolution of a large-scale ecosystem model is presented below.

Simulations have been performed using the simplest version of the ecosystem represented by only “transient” population (*n* = 1). Numerical study of the model has been conducted by means of NDSolve function with default settings in Mathematica tool (Wolfram Research, Inc., Mathematica, Version 11.0, Champaign, IL (2016)).

### The model for the self-development of a large ecosystem

In the model, the state of the ecosystem is characterized by the vector *X*(*t*) = (*x*_1_(*t*), …, *x*_*n*_ (*t*)), where *x*_*i*_(*t*) is the density of individuals, *i*—species with a self-reproduction potential at the moment *t*, *n*—number of species.

Further on, we will designate the totality of individuals with the potential for self-reproduction as biota. Let *W*_*i*_ denote the law of self-reproduction of biota of species *i*, and *Y*_*i*_ denote the law of the loss of self-reproduction potential, which we call the law of mortality. In general, the functional form of the laws of self-reproduction and mortality, *W*_*i*_ and *Y*_*i*_, is determined by the structure of the external environment and connections that exist between individuals of the ecosystem and their environment. That is, *W*_*i*_ and *Y*_*i*_ are functions that depend on time *t*, the density of species *X*(*t*), and a number of parameters *U* = {*u* }. Then, self-development of the ecosystem can be described by the following system of differential equations:

(1) \begin{eqnarray*} \frac{d{x}_{i}}{dt} \mathit{ = }{k}_{in,i}+{x}_{i}\cdot \left( {W}_{i}-{Y}_{i} \right) , i=1,\ldots ,n.\end{eqnarray*}

Here, *k*_*in*,*i*_ denotes the rate of the species *i* biota inflow from the outside into the modeling area.

### The model for the evolution of ecosystem parameters

In system Eq. (1), self-development is controlled by the laws of self-reproduction and mortality, *W*_*i*_ and *Y*_*i*_. We assume that evolution of an ecosystem is the process of changing the values of a subset of parameters *U* = {*u* }, which increases the adaptability of species.

Further on, parameters that change their values over time will be designated as evolving parameters. Without loss of generality, we can denote them *U* = {*u* }. Thus, parameters from *U* are not constants, but functions of time. As a result, in order to calculate system Eq. (1), it is necessary to know the form of functions *u*(*t*)∈*U*. To find their form, we derive differential equations that satisfy *u*(*t*) ∈*U*. First, we note that if value of the parameter equals *u*(*t*) at the current time, then its rate of change over time satisfies the general equation

\begin{eqnarray*} \frac{du}{dt} ={E}_{u}\cdot u, u\in U, \end{eqnarray*}

where, *E*_*u*_ is the rate at which the parameter *u* changes.

Without limiting generality, we assume that mutational variability of a particular parameter is a result of mutations that occur during reproduction of individuals of a single specific species. Let *σ*(*u*) denote this species. Further, we take into account that *E*_*u*_ value is formed as a result of two successive processes: (1) change in the value of the evolving parameter due to mutations that arise during reproduction of individuals of a particular species; (2) evolutionary selection of mutations that increase the adaptability of a given species. The probability of a change in the *u* value at the time *t* is denoted by *P*_*u*_, and by Ω_*u*_: the rate at which the parameter *u* changes under the influence of evolutionary selection. Accordingly, *E*_*u*_ = *P*_*u*_⋅Ω_*u*_ and the rate of evolutionary change *u*(*t*) satisfies the differential equation:

\begin{eqnarray*} \frac{du}{dt} ={P}_{u}\cdot {\Omega }_{u}\cdot u. \end{eqnarray*}

But, in general case, *P*_*u*_ and Ω_*u*_ are not constant values, but depend on the state of the ecosystem. Let us determined the type of *P*_*u*_ from the fundamental properties of living systems, that is the presence of inaccuracies, mutations, during genome replication in the process of self-reproduction. As a result, we obtain that *P*_*u*_ value is proportional to the specific intensity of reproduction of an individual:

\begin{eqnarray*}{P}_{u}={p}_{u}\cdot {W}_{\sigma (u)}, \end{eqnarray*}

where, *p*_*u*_ is the constant for mutation-associated change in the value of the evolving parameter *u*.

In general, *p*_*u*_ may not be a constant value, since replication accuracy may vary in the course of evolution, but for the purposes of our work, no further specification is required. Let us assume that *p*_*u*_ is a constant value.

To determine Ω_*u*_, we assume that at each time moment *t* the adaptability of the *σ*(*u*) species to the environmental conditions can be estimated by the value of the “fitness functional” *F*_*σ*(*u*)_. Let them be known for all species. Now we consider that not all mutations are useful. Only those mutations that make a positive contribution to increasing the value of the “fitness functional” *F*_*σ*(*u*)_ are being fixed in the genome. Hence we express Ω_*u*_ through *F*_*σ*(*u*)_. To do this, we note that in the absence of a mutation in the parameter *u* in a short time *h* the current value *F*_*σ*(*u*)_ changes by the value $h \frac{d{F}_{\sigma (u)}}{dt} (t,{U}^{{^{\prime}}}(t),u(t))$.

Here, *U*′(*t*) denotes the vector of evolving parameters with excluded parameter *u*. Imagine now that a mutation occurred at the time *t* that introduced a variation in the *u*(*t*) value and it became equal to *u*(*t*) + Δ*u*. Then at the moment *t* + *h* we have $h \frac{d{F}_{\sigma (u)}}{dt} (t,{U}^{{^{\prime}}}(t),u(t)+\Delta u)$.

From here we obtain an increase in the “fitness functional” *F*_*σ*(*u*)_, which is achieved due to the mutational change in the evolving parameter *u*_*j*_:

\begin{eqnarray*}\begin{array}{@{}l@{}} \displaystyle {\Omega }_{u}={\mathrm{lim}}_{\Delta u\rightarrow 0}li{m}_{h\rightarrow 0} \left[ \frac{h \left( \frac{d{F}_{\sigma (u)}}{dt} \left( t,{U}^{{^{\prime}}}(t),u+\Delta u \right) - \frac{d{F}_{\sigma (u)}}{dt} \left( t,{U}^{{^{\prime}}}(t),{u}_{i,j} \right) \right) }{h\Delta u} \right] \\ \displaystyle ={\mathrm{lim}}_{\Delta u\rightarrow 0} \left[ \frac{ \frac{d{F}_{\sigma (u)}}{dt} \left( t,{U}^{{^{\prime}}}(t),u+\Delta u \right) - \frac{d{F}_{\sigma (u)}}{dt} \left( t,{U}^{{^{\prime}}}(t),{u}_{i,j} \right) }{\Delta u} \right] \\ \displaystyle = \frac{\partial }{\partial u} \left( \frac{d{F}_{{}_{\sigma (u)}}}{dt} \right) . \end{array} \end{eqnarray*}

As a result, for each parameter *u*, we obtain an evolution equation of the general form

(2) \begin{eqnarray*} \frac{du}{dt} \mathit{ = }{p}_{u}\cdot u{W}_{\sigma (u)}\cdot \frac{\partial }{\partial u} \left( \frac{d}{dt} {F}_{\sigma (u)} \right) .\end{eqnarray*}

Note that Eq. (2) does not include the “fitness functional” *F*_*σ*(*u*)_, but its total time derivative $ \frac{d{F}_{\sigma (u)}}{dt} $.

That is the difference between Eq. (2) and analogous equation previously used in the models of the evolution of ecosystems (Sznajd-Weron & Weron, 2001). The set of equations Eq. (2) taken for all evolving parameters *u* ∈ *U* represents a model for the evolution of ecosystem parameters. Together with system (1), they represent a model for the evolutionary self-development of a large ecosystem. For clarity, we shall write it down

(3) \begin{eqnarray*} \left\{ \begin{array}{@{}l@{}} \displaystyle \frac{d{x}_{i}}{dt} ={k}_{in,i}+{x}_{i}\cdot \left( {W}_{i}-{Y}_{i} \right) , i=1,\ldots ,n,\\ \displaystyle \frac{du}{dt} \mathit{ = }{p}_{u}u\cdot {W}_{\sigma (u)}\cdot \frac{\partial }{\partial u} \left( \frac{d}{dt} {F}_{\sigma (u)} \right) , u\in U. \end{array} \right. \end{eqnarray*}

### The law of biota self-reproduction

Let us assume that individuals of a “transient” population have a mixed (sexual and asexual) type of reproduction and sexual reproduction requires the meeting of two individuals. Then we describe the specific self-reproduction rate of a transit type biota by the classical logistic model, which assumes a simple positive feedback between diversity and its growth rate (parents—more descendants), and negative feedback based on the limited ecological space available for reproduction:

(4) \begin{eqnarray*}W= \left( {k}_{aSx}+{k}_{Sx}x \right) \left( 1- \frac{x}{C} \right) \end{eqnarray*}

In Eq. (4) *k*_*aSx*_ and *k*_*Sx*_ are rate constants for asexual and sexual reproduction, factor *x* corresponds to the sexual reproduction, factor (1 − *x*/*C*) describes the negative effect of biota density on the self-reproduction rate, *C*—maximum possible of biota density.

We used a logistic model because density dependence is a general tendency and fundamental principle of population ecology with potentially regulatory effects on population growth whereby the population has a propensity to increase when small and decrease when large (Hixon, Pacala & Sandin, 2002; Knape & De Valpine, 2012).

Also, according to AV Markov, hypothesis that the dynamics of the Phanerozoic marine biota calculated by traditional methods (without amendments) adequately reflects real changes in biodiversity has not been unproved and remains the most convenient and reliable basis for meaningful biological interpretations (Markov & Korotaev, 2007, p. 4).

The specific rate of mortality of the biota of a transit type we describe by the following function:

(5) \begin{eqnarray*}Y={k}_{d}+D\cdot H.\end{eqnarray*}

In Eq. (5), *k*_*d*_ denotes the specific rate of mortality, which is determined by environmental conditions. The second addendum (*D*⋅*H*) sets the specific rate of mortality, which is determined by the internal laws of the ecosystem’s functioning. We write it as multiplication of the rate constant for degradation *D* by the function *H*, which determines the mechanism of the influence of biota density on the mortality of individuals of the “transient” population. Assume that *H* is a bounded monotonically increasing function of the biota density. This assumption is implemented through the function:

(6) \begin{eqnarray*}H= \frac{{k}_{d}\cdot x\cdot {e}^{ \frac{x}{{K}_{E}} -1}}{{K}_{D}+x\cdot {e}^{ \frac{x}{{K}_{E}} -1}} .\end{eqnarray*}

In Eq. (6) $r={e}^{ \frac{x}{{K}_{E}} -1}$denotes the coefficient of “auto-aggression”, *K*_*D*_ and *K*_*E*_ –parameters. The coefficient of specific “auto-aggression” in *H*(*x*) describes density-induced stress, which decreases the rate of reproduction (Ostfeld, Canham & Pugh, 1993; Wauters et al., 2004; Zhao et al., 2019) that may occur irrespective of taxon, life history and body size (Odden et al., 2014). In the model its value is monotonic, nonlinear, increases with increasing biota density *x* from 0 to 1: at zero density there is no “aggression”—*H*(0) = 0; at high density the coefficient of specific “aggression” tends to 1 − *H*(∞) = 1.

### General form of the model of evolution of a “transient” population

Thus, the model (3) describes evolution as a process of ecosystem self-development (population of a “transit” type), during which there is a local increase in the adaptability of its individuals to the environmental conditions due to mutational variability and natural selection. The model has the following characteristics:

• mixed (asexual/sexual) type of reproduction,
• logistic law of reproduction *W*,
• exponentially rational law *H* of the influence of population density on the average life span of an individual,
• two evolving parameters *C* and *D*,
• function of adaptability *F*_*fitness*_ = *W*,
• influx or migration of live biota from the outside, *k*_*in*_.

Why adaptability function *F*_*fitness*_ = *W*?

*A priori*, there are two natural measures for assessing the adaptation degree of a “transient” population to habitat conditions: *W* reflects the relative, specific efficiency of its self-reproduction, and *xW*—the absolute rate of its reproduction. We called them “fitness functionals”, *F*_1_ and *F*_2_, respectively. We have previously shown (Likhoshvai et al., 2017) that *F*_1_ “fitness functional” allows to describe both the stable functioning of populations with asexual reproduction, for example, cyanobacteria that persist and develop for at least two billion years (Butterfield, 2007), and cyclic dynamics of the Phanerozoic marine biota (Rohde & Muller, 2005; Melott, 2008; Lieberman & Melott, 2007; Melott & Bambach, 2014; Melott & Bambach, 2017; Roberts & Mannion, 2019), which is characterized by sexual reproduction. Based on this, we assume that in model (3) *F* ≡*W*.

Further, if we write Eqs. (2) of model (3) for each evolving parameter, reproduction parameter *C* and mortality parameter *D*, and assume *n* = 1, we have the following evolutionary model of a “transient” population:

(7) \begin{eqnarray*} \left\{ \begin{array}{@{}l@{}} \displaystyle \frac{d}{dt} x={k}_{in}+x \left( W-Y \right) ,\\ \displaystyle \frac{d}{dt} C={p}_{C}WC \frac{\partial }{\partial C} \left( \frac{d}{dt} F \right) ,\\ \displaystyle \frac{d}{dt} D={p}_{D}WD \frac{\partial }{\partial D} \left( \frac{d}{dt} F \right) , \end{array} \right. \end{eqnarray*}

where *x*(*t*) is the density of transitive individuals and *W* and *Y* are the laws of self-production and mortality, respectively, which are determined based on the Eqs. (4) and (5). The parameters *p*_*C*_ and *p*_*D*_ stand for the rate constants for mutation-associated change in the values of the “reproduction” parameter *C* and “mortality” parameter *D*. There is no doubt that parameters *p*_*C*_ and *p*_*D*_ also changed values during evolution. But in the present study, we consider them to be constant. Since $ \frac{d}{dt} F={W}_{x}{x}_{t}+{W}_{C}{C}_{t}$, partial derivatives $ \frac{d}{dt} F$ with respect to the parameters *C* and *D* have the following form:

\begin{eqnarray*}\begin{array}{@{}l@{}} \displaystyle \frac{\partial }{\partial C} \left( \frac{d}{dt} F \right) = \frac{\partial }{\partial C} \left[ {W}_{x}{x}_{t}+{W}_{C}{C}_{t} \right] ,\\ \displaystyle \frac{\partial }{\partial D} \left( \frac{d}{dt} F \right) = \frac{\partial }{\partial D} \left[ {W}_{x}{x}_{t}+{W}_{C}{C}_{t} \right] , \end{array} \end{eqnarray*}

where

\begin{eqnarray*}\begin{array}{@{}l@{}} \displaystyle \frac{d}{dx} W={W}_{x}={k}_{Sx}- \frac{{k}_{aSx}+2{k}_{Sx}x}{C} ,\\ \displaystyle \frac{d}{dC} W={W}_{C}= \left( {k}_{aSx}+{k}_{Sx}x \right) \frac{x}{{C}^{2}} , \frac{\partial }{\partial C} \left( \frac{d}{dC} W \right) ={W}_{CC}=-2 \left( {k}_{aSx}+{k}_{Sx}x \right) \frac{x}{{C}^{3}} ,\\ \displaystyle \frac{\partial }{\partial C} \left( \frac{d}{dx} W \right) ={W}_{xC}= \left( {k}_{aSx}+2{k}_{Sx}x \right) \frac{1}{{C}^{2}} ,\\ \displaystyle H= \frac{{k}_{d}\cdot x\cdot {e}^{ \frac{x}{{K}_{E}} -1}}{{K}_{D}+x\cdot {e}^{ \frac{x}{{K}_{E}} -1}} , \frac{d}{dx} H={H}_{x}={k}_{d}\cdot {K}_{D} \frac{ \left[ \left( 1+ \frac{x}{{K}_{E}} \right) \cdot {e}^{ \frac{x}{{K}_{E}} -1} \right] }{{ \left( {K}_{D}+x\cdot {e}^{ \frac{x}{{K}_{E}} -1} \right) }^{2}} . \end{array} \end{eqnarray*}

Let us find the solution of the functions *C*_*t*_ and *D*_*t*_ (the derivation is given in the Supplementary Information 1). After performing the calculations, we arrive at the following model of evolution:

(8) \begin{eqnarray*}\begin{array}{@{}l@{}} \displaystyle \left\{ \begin{array}{@{}l@{}} \displaystyle {x}_{t}={k}_{in}+x \left( W-Y \right) \\ \displaystyle {C}_{t}=-{p}_{C}W\sum _{i=1}^{\infty }i \frac{{u}_{i}+Y{w}_{i}}{{C}^{i}} \\ \displaystyle {D}_{t}={p}_{D}WDH\sum _{i=0}^{\infty } \frac{{w}_{i}}{{C}^{i}} \end{array} \right. \\ \displaystyle F=W,Y=DH+{k}_{d},\\ \displaystyle W= \left( {k}_{aSx}+{k}_{Sx}x \right) (1- \frac{x}{C} ), \frac{d}{dx} W={W}_{x}={k}_{Sx}- \frac{{k}_{aSx}+2{k}_{Sx}x}{C} ,\\ \displaystyle \frac{d}{dC} W={W}_{C}= \left( {k}_{aSx}+{k}_{Sx}x \right) \frac{x}{{C}^{2}} , \frac{d}{dC} \left( \frac{d}{dC} W \right) ={W}_{CC}=-2 \left( {k}_{aSx}+{k}_{Sx}x \right) \frac{x}{{C}^{3}} ,\\ \displaystyle \frac{d}{dC} \left( \frac{d}{dx} W \right) ={W}_{xC}= \left( {k}_{aSx}+2{k}_{Sx}x \right) \frac{1}{{C}^{2}} ,\\ \displaystyle H= \frac{{k}_{d}\cdot x\cdot {e}^{ \frac{x}{{K}_{E}} -1}}{{K}_{D}+x\cdot {e}^{ \frac{x}{{K}_{E}} -1}} , \frac{d}{dx} H={H}_{x}={k}_{d}\cdot {K}_{D} \frac{ \left[ \left( 1+ \frac{x}{{K}_{E}} \right) \cdot {e}^{ \frac{x}{{K}_{E}} -1} \right] }{{ \left( {K}_{D}+x\cdot {e}^{ \frac{x}{{K}_{E}} -1} \right) }^{2}} ,\\ \displaystyle {u}_{0}= \left( {k}_{Sx} \right) \left( {k}_{in}+x \left( \left( {k}_{aSx}+{k}_{Sx}x \right) \right) \right) ,\\ \displaystyle {u}_{1}=- \left[ {k}_{in} \left( {k}_{aSx}+2{k}_{Sx}x \right) +x \left( {k}_{aSx}+{k}_{Sx}x \right) \left( {k}_{aSx}+3{k}_{Sx}x \right) \right] ,\\ \displaystyle {u}_{2}={x}^{2} \left( {k}_{aSx}+2{k}_{Sx}x \right) \left( {k}_{aSx}+{k}_{Sx}x \right) ,\\ \displaystyle {u}_{3}=-{p}_{C}x{ \left( {k}_{aSx}+{k}_{Sx}x \right) }^{2}{u}_{1},\\ \displaystyle {u}_{i}={p}_{C}x{ \left( {k}_{aSx}+{k}_{Sx}x \right) }^{2} \left[ \left( i-3 \right) x{u}_{i-3}- \left( i-2 \right) {u}_{i-2} \right] , i=4,\ldots \\ \displaystyle {w}_{0}=-{k}_{Sx}x,{w}_{1}= \left( {k}_{aSx}+2{k}_{Sx}x \right) x,{w}_{2}=0,{w}_{3}=-{p}_{C}x{ \left( {k}_{aSx}+{k}_{Sx}x \right) }^{2}{w}_{1},\\ \displaystyle {w}_{i}={p}_{C}x{ \left( {k}_{aSx}+{k}_{Sx}x \right) }^{2} \left[ \left( i-3 \right) x{w}_{i-3}- \left( i-2 \right) {w}_{i-2} \right] , i=4,\ldots \end{array}\end{eqnarray*}

Numerical calculations of the model Eq. (8), depending on the type of reproduction of the transit population (*k*_*aSx*_, *k*_*Sx*_) and the values of the evolutionary parameters (*p*_*C*_, *p*_*D*_) is shown below (see also Supplementary Information 2).

## Results

### Cyclic and intermittent dynamics in the model of evolution of a “transient” population

**Figure 1:**
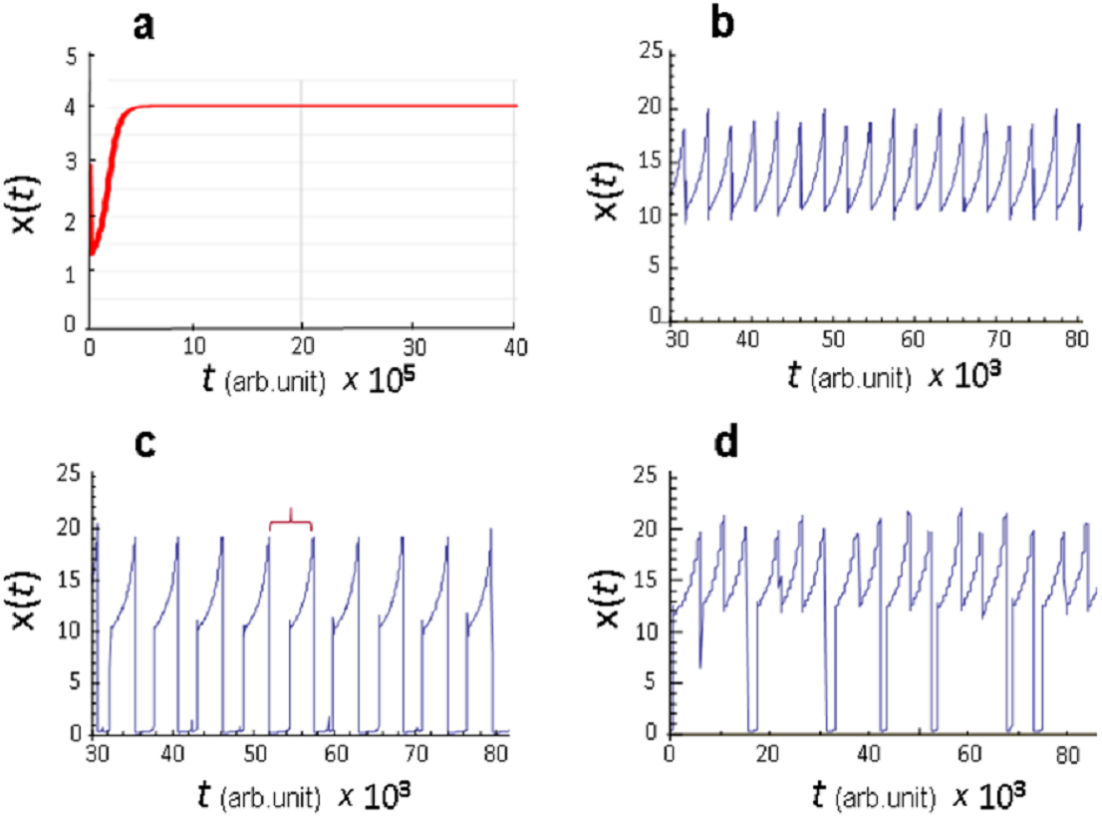
Dynamic modes of functioning of the model Eq. (8). (A) Stationary state at *k*_*Sx*_ = 0 (asexual type of reproduction); (B–D) oscillatory regime at *k*_*aSx*_ = 0 (sexual type of reproduction), values of the parameters *p*_*C*_ and *p*_*D*_ are given in conventional units: (B) *p*_*C*_ = 0.0025, *p*_*D*_ = 0.045, (C) *p*_*C*_ = 0.0025, *p*_*D*_ = 0.035, (D) *p*_*C*_ = 0.0015, *p*_*D*_ = 0.02111. Red bracket marks one oscillatory cycle of the parameter *x*(*t*).

The key information provided by numerical calculations was observation that only stationary dynamics *x*(*t*) is observed in model Eq. (8) at *k*_*Sx*_=0 (no sexual type of reproduction) (Fig. 1A), that is, constancy (stasis) of diversity is established in the course of evolution of a “transient” population. At the same time, at *k*_*aSx*_=0 (no asexual type of reproduction) in model Eq. (8) for certain values of the parameters *p*_*C*_ and *p*_*D*_, which are the rate constants for mutation-associated change in the values of the reproduction parameter *C* and mortality parameter *D*, undamped oscillatory dynamics of *x*(*t*) variation was observed (Figs. 1B and 1C). Moreover, this dynamics became chaotic at certain values of the parameters *p*_*C*_ and *p*_*D*_ (Fig. 1D).

It is significant that oscillatory dynamics is generated due to only four factors: (1) presence of sexual reproduction; (2) negative impact of population density on the rates of reproduction and mortality of biota individuals; (3) genetic variation caused by mutations that occur during replication; (4) evolutionary selection directed towards increasing the adaptability of the individuals to habitat conditions.

Therefore, the results presented in Figs. 1B–1D already allow us to explain the oscillatory (cyclic) dynamics of changes in species diversity observed in marine biota in the last 500 million years without resorting to external influences.

However, the calculations obtained allow us to move forward. Namely, the same factors can explain such aspects of punctuated evolution observed in the fossil record as extinction catastrophes, rapid growth phases and stasis phases of species diversity. Indeed, analysis of the model revealed that in the oscillatory kinetics presented in Figs. 1C and 1D, qualitatively different phases of the *x*(*t*) value evolution are clearly distinguished. Let us consider them in more detail. Any arbitrary interval including a full cycle, similar to the one marked with a red bracket in Fig. 1C, is reasonable for this. Such cycle presents a typical scenario of the evolutionary turn of ecosystem development, in the sense that qualitative properties observed over a given time interval are reproduced in the course of evolution.

**Figure 2:**
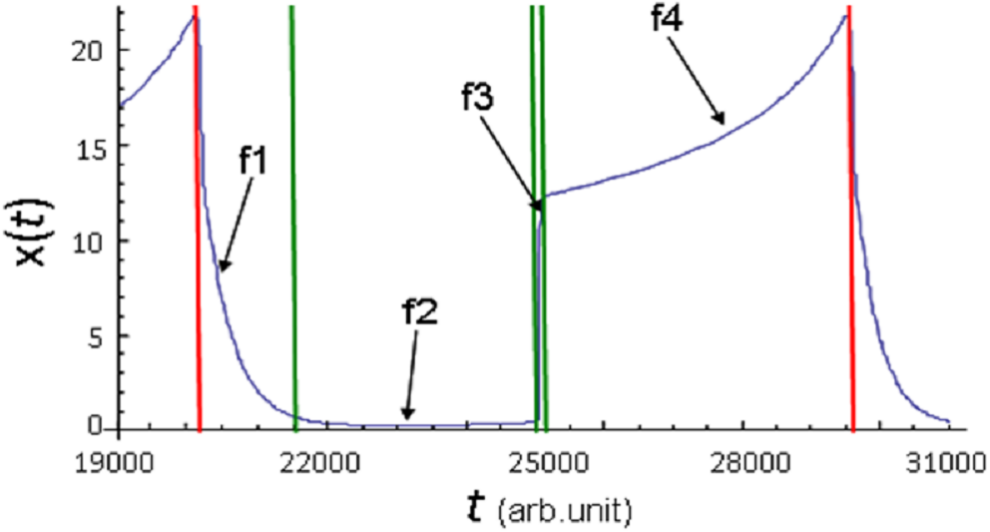
Phases of *x*(*t*) parameter evolution demonstrated onthe example of one full cycle of oscillation of its values. Calculation of model Eq. (8) at *p*_*C*_ = 0.0015, *p*_*D*_ = 0.0205(*C*). Red vertical lines indicate the boundaries of the cycle, green vertical lines indicate the boundaries of the phases within the cycle.

Four phases of the parameter *x*(*t*) evolution derived from the model Eq. (8) calculation at *p*_*C*_ = 0.0015 and *p*_*D*_ = 0.0205 are shown in Fig. 2. It can be clearly seen that f1 phase is characterized by a fast, on the scale of calculations, almost vertical decrease in *x*(*t*) to almost zero. This phase qualitatively corresponds to the degree of mass extinctions observed on Earth at least three times: 65, 250, 370 million years ago (see Fig. 1C in: Rohde & Muller, 2005). f2 phase is characterized by the low *x*(*t*) value. This phase qualitatively corresponds to the stages of life development that follow mass extinctions. This phase is characterized by a very low biodiversity level and very slow growth over a certain time interval. Then follows the phase of increasing species diversity, which can be divided into two stages. *x*(*t*) value rapidly increases during the first stage (Fig. 2, f3 phase). f3 phase of oscillatory dynamics qualitatively corresponds to the stages of explosive growth in the terrestrial biodiversity. The second stage is characterized by a high *x*(*t*) value, which continues to increase slowly (Fig. 2, f4 phase). f4 phase qualitatively corresponds to those stages of life development on Earth, in which a great variety of life forms and a relatively low growth rate are observed. Usually such stages of evolution are described as stasis.

In the model, losses and gains in species diversity inevitably repeat an unlimited number of times with approximately equal time intervals.

It should be noted that, depending on the calculation, f4 phase may consist of one or more segments of a gradual change in the *x*(*t*) value. In the case of several segments (see Fig. 1C), there are areas with a rapid decrease in *x*(*t*) value between them. However, the minimum achieved in these areas is much higher than the minima achieved in f1 phase; therefore, the observed decrease in *x*(*t*) can be interpreted as local catastrophe. It corresponds to the mild evolution that was shown in the calculation of model Eq. (8) with parameter values *p*_*C*_ = 0.0025, *p*_*D*_ = 0.045 (see Fig. 1B). Here, all evolutionary development consists of only f4 phase realization, in which oscillations of the parameter *x*(*t*) occur.

Thus, the modeling results have shown that if the efficiency of reproduction and mortality in a population depends on its density and the most adapted individuals, the genetic diversity of which is a result of genome replication errors during self-reproduction, are being selected, then these conditions are *sufficient* for the formation of cyclical intermittent dynamics of biodiversity in a living system with sexual type of reproduction.

Therefore, both cyclic and intermittent dynamics of changes in the diversity of organisms with sexual reproduction that make up the Earth’s global ecosystem observed in the last 500 million years may well be explained solely by internal laws of self-development with no impact of external factors.

The question arises—what properties of the system Eq. (8) determine the nature of the evolution of the *x*(*t*) curve?

### Global extinction in the history of life as a bistability phenomenon

**Figure 3:**
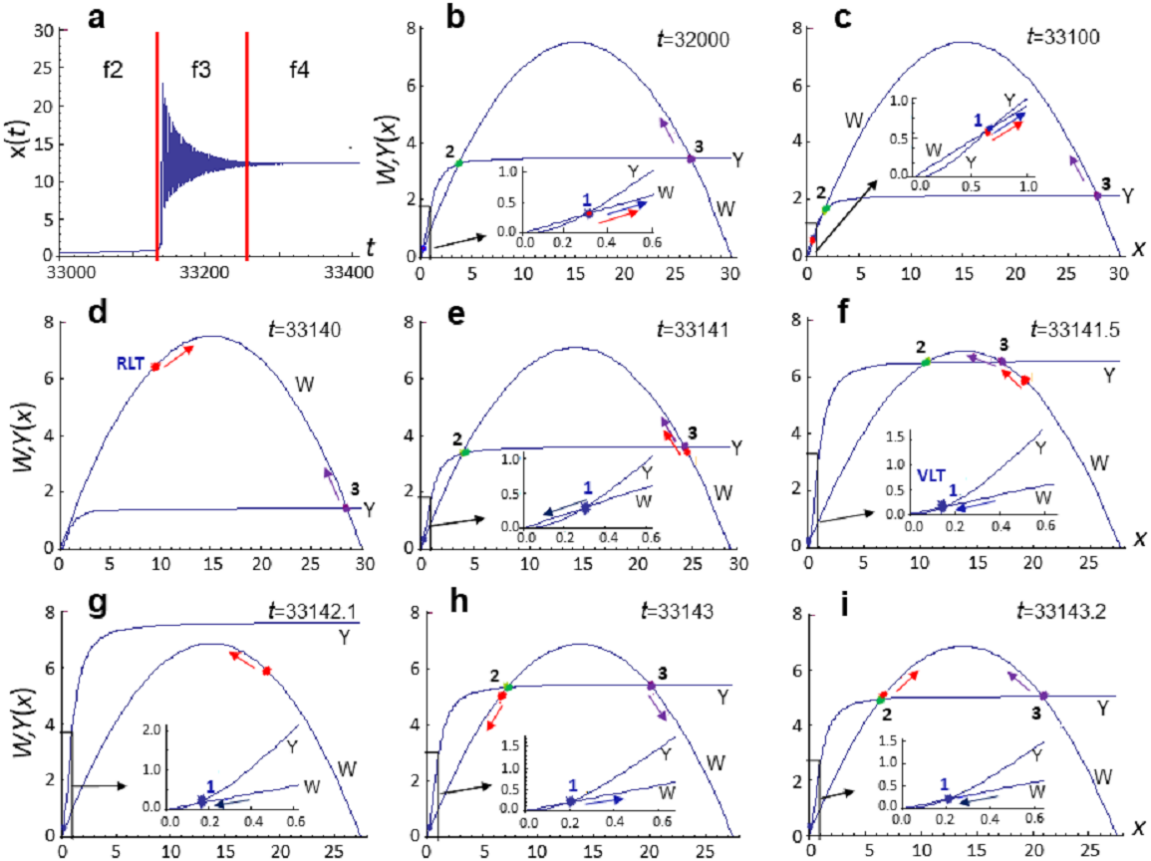
Diagrams of the functions *W* and *Y* at different stages of the evolution of system Eq. (8). Calculation of model Eq. (8) at *p*_*C*_ = 0.0015, *p*_*D*_ = 0.02111. (A) Analyzed section of the *x*(*t*) curve, vertical lines indicate the phase boundaries of the analyzed section. (B)–(I) Diagrams of the functions *W* and *Y*, constructed at the fixed time moment *t*. Parameter values (in conventional units): (B) *t* = 32,000, *C* = 30.0820, *D* = 3.5, *x*_*min*_ = 0.3098, *x*_*mdl*_ = 3.72, *x*_max_ = 26.045, *x* = 0.3096; (C) *t* = 33100, *C* = 30.0834, *D* = 2.1261, *x*_min_ = 0.661, *x*_*mdl*_ = 1.64, *x*_max_ = 27.78, *x* = 0.6478; (D) *t* = 33140, *C* = 30.1132, *D* = 1.4357, *x*_min_—no value, *x*_*mdl*_—no value, *x*_max_ = 28.6, *x* = 9.01; (E) *t* = 33141, *C* = 28.5246, *D* = 3.6068, *x*_min_ = 0.3, *x*_*mdl*_ = 3.93(2), *x*_max_ = 24.3, *x* = 24.72; (f) *t* = 33141.5, *C* = 27.6767, *D* = 6.5723, *x*_min_ = 0.15, *x*_*mdl*_ = 10.5, *x*_max_ = 17.04, *x* = 19.3307; (G) *t* = 33142.1, *C* = 27.46, *D* = 7.59, *x*_min_ = 0.13, *x*_*mdl*_—no value, *x*_max_—no value , *x* = 18.6191. (H) *t* = 33143.0, *C* = 27.5, *D* = 5.43, *x*_min_ = 0.19, *x*_*mdl*_ = 7.23, *x*_max_ = 20.1, *x* = 6.561; (I) *t* = 33143.2, *C* = 27.51, *D* = 5.06, *x*_min_ = 0.2, *x*_*mdl*_ = 6.46, *x*_max_ = 20.84, *x* = 6.47. Point (1)—stable stationary state *x*_min_, point (2)—unstable stationary state *x*_*mdl*_, point (3)—stable stationary state *x*_max_, red point—current *x*(*t*) value. Colored arrows indicate the direction of evolution (change) of parameters.

In Eq. (8), the “transient” biota density determined by the *x*(*t*) variable. The value of *x*(*t*) parameter in model Eq. (8) at each moment in time depends on the ratio between the rates of reproduction and mortality described by the functions *W* and *Y*. If we plot these functions at different times of evolution *x*(*t*), then we can note that curves *W* and *Y* can intersect up to three times (see Figs. 3B–3I). Each intersection corresponds to a stationary state, which can have the first equation of the dynamical system Eq. (8) at the fixed values of the parameters *C* and *D*.

Let us analyze the specific calculations of model Eq. (8) that are implemented at *p*_*C*_ = 0.0015, *p*_*D*_ = 0.02111 in the interval *t*∈[32000,33400] conv. time units in more detail. Transition from phase f2 to phase f3 and then to phase f4 occurs in the evolutionary cycle of biota development within the specified interval (see Fig. 3A). That is, it qualitatively corresponds to the stages of life development that follow global extinction.

Calculation of the model Eq. (8) at *t* = 32,000 is shown in Fig. 3B. At this time moment *x*(*t*)∼0.3096 and the first equation of system Eq. (8) has two stable stationary states: *x*_min_ ∼ 0.3 and *x*_max_ ∼ 26 with an unstable stationary state *x*_*mdl*_ between them. Since *x*_min_∼*x*(*t*)<*x*_*mdl*_ at the given time moment, current *x*(*t*) position lies in the attraction region of the stationary state *x*_min_. This means that if we fix values of *C* and *D* at the given time moment, *x* (*t*) will tend to the stationary state *x*_min_. Such scenario is interpreted as a state of minimum species richness (corresponds to f2 phase). The fact that at the same time, system Eq. (8) has another stable stationary state *x*_max_ does not affect the state of the system, since *x*(*t*) value lies outside the region of its attraction and the system cannot get into it without external influences. Therefore, we can assume that at time *t* = 33000, the *x*_min_ stationary state is manifested, and the *x*_max_ stationary state is not manifested.

At every current moment, system Eq. (8) is in a position that is in the attraction area of the manifested stationary state, whose parameters determine the state of the system, and it is the increase in the integral adaptiveness of this system that drives the evolutionary progress.

In the model, this is achieved via mutational change in the *C* and *D* values and by fixing only those values that lead to an increase in adaptability. Indeed, the following calculation at *t* = 33100 (Fig. 3C) shows that, in the course of time, system Eq. (8) evolves toward an increase in the number and diversity of biota—*x*(*t*) value increases, while the attraction area of the stationary state *x*_min_ decreases; *x*_min_ and *x*_*mdl*_ stationary states converge, merge and disappear at some point within the interval *t*∈[33100,33140], so that we observe only one stationary state *x*_max_ at *t* = 33140 (Fig. 3D). Current *x*(*t*) value falls into the attraction area of this stationary state.

Since value of the stationary state *x*_max_ >  > *x*(*t*), an explosive increase in *x*(*t*) value is observed and *x*(*t*) value increases by an order of magnitude (Figs. 3D and 3E) and becomes larger than *x*_max_ (Figs. 3E and 3F) over a very short period of time (∼ per one conventional unit). As a result, f2 phase changes to f3 phase, during which the *x*(*t*) value reaches a new quantitative level. This event is accompanied by oscillations of *x*(*t*) with significant amplitude and small period (Fig. 3A), which arise as a result of the instability of the stationary state *x*_max_ (Fig. 3G). However, after a certain number of oscillations (Figs. 3H and 3I), the stability of the stationary state *x*_max_ is restored, the system goes into phase 4 and further evolution proceeds in the attraction area of the stationary state *x*_max_.

**Figure 4:**
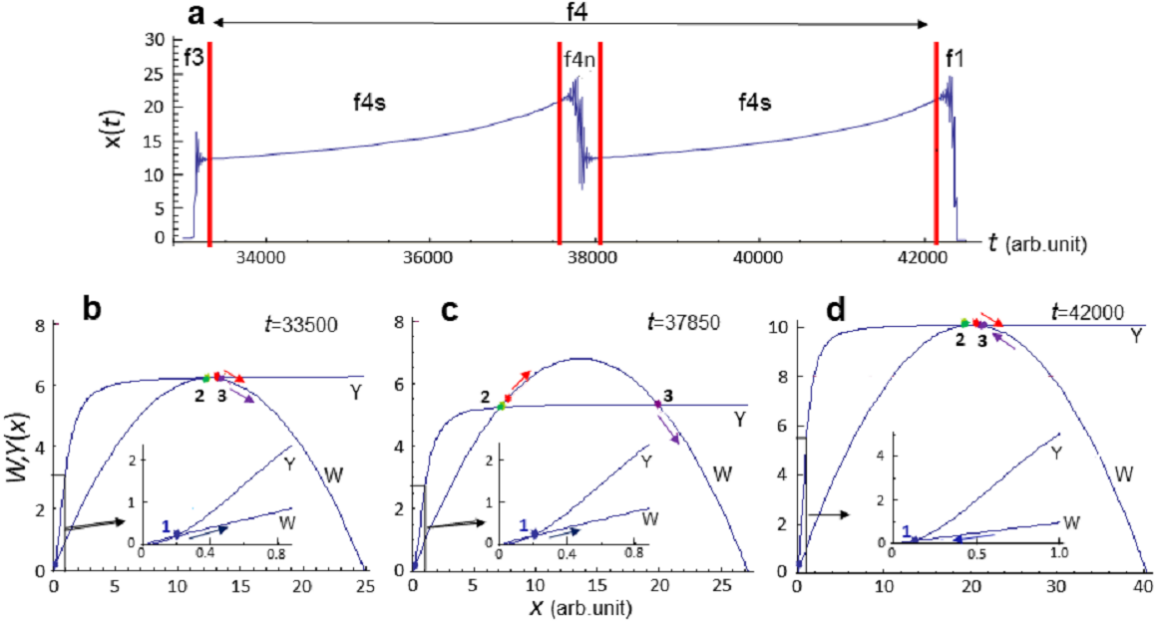
Diagrams of the functions *W* and *Y* at f4 phase of the evolution of system Eq. (8). Calculation of model Eq. (8) at *p*_*C*_ = 0.0015, *p*_*D*_ = 0.02111. (A) Analyzed section of the *x*(*t*) curve, vertical lines indicate the phase boundaries of the analyzed section. (B)–(D) Diagrams of the functions *W* and *DH* constructed at fixed time moment *t*. Parameter values (in conventional units): (B) *t* = 33500, *C* = 24.89, *D* = 6.26, *x*_min_ = 0.16, *x*_*mdl*_ = 12.23, *x*_max_ = 12.49, *x* = 12.47; (C) *t* = 37850, *C* = 27.22, *D* = 5.35, *x*_min_ = 0.19, *x*_*mdl*_ = 7.1, *x*_max_ = 19.92, *x* = 7.67; (D) *t* = 42000, *C* = 40.38, *D* = 10.12, *x*_min_ = 0.1, *x*_*mdl*_ = 20.43, *x*_max_ = 20.43, *x* = 20.28. Point (1)—stable stationary state *x*_min_, point (2)—unstable stationary state *x*_*mdl*_, point (3)—stable stationary state *x*_max_, red point—current *x*(*t*) value. Colored arrows indicate the direction of evolution (change) of parameters.

Subsequently, evolution of the system Eq. (8) proceeds within the f4 phase, which can be characterized as a stasis phase (Fig. 4). During phase f4, *x* is quite high and, in the framework of the analysis, increases approximately twofold (Figs. 4B and 4D), and evolution proceeds in the attraction area of the stationary state *x*_max_ (Figs. 4B–4D). f4 phase is the longest and is not qualitatively homogeneous. It is divided into subphases called f4s—the monotonic growth of *x*(*t*) value, and f4n, which can be interpreted as local extinction. However, the exact number of subphases cannot be predicted due to the chaotic dynamics of evolution (Fig. 1D).

The exact duration of each phase cannot be predicted with a set of values *p*_*C*_ = 0.0015, *p*_*D*_ = 0.02111, since the dynamics observed in model Eq. (8) is chaotic (Fig. 1D).

Thus, we have demonstrated that such features of punctuated evolution observed in the fossil record as global extinctions and phases of rapid growth and biodiversity stasis are a reflection of the emergence of bistability (two distinct stable states) in a self-organizing system, which is the Earth’s biota. Transition from one stable state to another is accompanied by a rapid change in the parameters of the system, which may reflect the uneven pace of evolution observed in phylogenetic studies (Nichol, Rowe & Fitch, 1993; Pagel, Venditti & Meade, 2006; Wolf et al., 2006; Palmer et al., 2012). The reason for the transition from one state to another is the selection of the most adapted individuals, the genetic diversity of which is a result of genome replication errors during self-reproduction.

## Discussion

Living systems are part of dynamic systems, so mathematical modeling can be used for discovering fundamental laws of living systems. An attempt to explain the dynamics of biodiversity observed in the Earth’s fossil record solely by external factors means ignoring the possibility that a dynamic system, such as Earth’s biota, has internal mechanisms that determine its functioning mode.

It is true that individual episodes of global extinctions in the history of the planet coincide with the influence of external factors (Alvarez et al., 1981; Wignall et al., 2009; Courtillot & Fluteau, 2010; Schulte et al., 2010; Rampino et al., 2017; Smolarek-Lach et al., 2019).

However, we asked a question—can the phenomenon of rapid evolution and the associated periodicity of global extinctions be explained only by internal laws?

In a number of theoretical studies, the existence of discontinuity and phasing in the fossil record was considered as a consequence of a priori existing regulatory feedback loops - negative and positive, the combination of which led to system instability (Robertson, 1991; Seaborg, 1999). This property of feedback regulatory loops has been noted long ago and was demonstrated in models of biological systems at various levels of their organization (Mackey & Glass, 1977; Decroly & Goldbeter, 1982; Martinez de la Fuente et al., 1996; Goldbeter et al., 2001; Likhoshvai et al., 2014; Likhoshvai et al., 2015; Likhoshvai et al., 2016; Likhoshvai, Golubyatnikov & Khlebodarova, 2020; Harish & Hansel, 2015; Kogai et al., 2017; Khlebodarova et al., 2017; Khlebodarova et al., 2018; Khlebodarova, Kogai & Likhoshvai, 2020).

However, this is not the only mechanism that can lead to instability in nonlinear dynamic systems. It was found that the very fact that the system has a tendency for self-development at a certain stage of its evolution can act as destabilizing factor.

It has been previously demonstrated that the simplest self-reproduced system where two genes orchestrate its development cycle can have bistable behaviour during the evolution and the second emerged phenotype was called as latent (Likhoshvai & Matushkin, 2000; Likhoshvai & Matushkin, 2004). It turned out this phenotype really exists but occurs at a very low frequency. The biological example of latent phenotype is persistent cells (Balaban et al., 2004; Shah et al., 2006). We showed that phenotypic switch (or transition from one metabolic state to another) leading to their emergence can be related to the bistability and underlie the biodiversity (Khlebodarova & Likhoshvai, 2018; Khlebodarova & Likhoshvai, 2019).

We suggested that the bistable switch-like behaviour may play a similar role on higher hierarchical organization levels of living systems and cause uneven paces of the evolution during changeable environmental conditions.

In this paper, we have shown that evolving ecosystems with sexual reproduction are likely to have bistability and transition from one to another can be described as rapid evolution. Moreover, change in the dynamic parameters of the system Eq. (8) during transition from one stationary state to another can underlie the observed uneven evolutionary rate, which has been convincingly demonstrated at a molecular level (Nichol, Rowe & Fitch, 1993; Pagel, Venditti & Meade, 2006; Wolf et al., 2006; Palmer et al., 2012).

The data obtained support the assumption that the bistability can be a feature of the evolutionary process in general, while mechanisms of its implementation depend on the organization level. We assume that the emergence of bistability may be due to nonlinear regulation and complexity of mechanisms of heredity transmission via transcription/translation on the cellular level and sexual reproduction on the population level.

Bistability implies a capability for changes in the biodiversity, which a priory occur on different time scales depending on the organization level of the living system. Estimates of the lifetime of species, genus and periodicity of global extinctions implicitly support this suggestion: ∼0.5 and ∼5.9 million years for the species and genus lifespan, respectively (Gingerich, 1976; Severtsov, 1990), and ∼60 million years for periodicity that has been seen in the Earth’s fossil record (Rohde & Muller, 2005; Melott, 2008; Lieberman & Melott, 2007; Lieberman & Melott, 2012).

Based on this we assume that discontinuity and associated periodicity in the development of Earth’s biota are also a reflection of the emergence of bistability in a self-organizing system.

Linking the obtained results with our previous study (Likhoshvai et al., 2017), we came to the conclusion that adaptation to the prevailing external conditions by means of gradual accumulation of mutations (the process of evolution), which ensures the increase of both biota density and diversity, inevitably leads to the repetition of the scenario - another catastrophe followed by biodiversity change, which we observe in the Earth’s fossil record.

We explain global extinctions phases and stasis of species by the influence of the same factors. The theory presented does not exclude that at certain points in time the Earth’s biota was subjected to powerful external influences, which had a significant impact on its further development, which, undoubtedly, was reflected in specific paleontological findings. However, according to our theory, even if known external influences in the Earth’s history would not have happened, the periodic phenomena of global extinctions of species followed by appearance of new types would inevitably occur, since such scenario is dictated only by the internal laws of functioning of ecosystems. To this, we can add that some researchers classify current biodiversity crisis as next mass extinction. This opinion is based on the estimate of the extinction rate of animals and plants in the present period, which is comparable to that in the periods of mass extinctions estimated from paleontological data (Barnosky et al., 2011; Ceballos et al., 2015).

## Conclusion

In this article, we argue that global extinctions and phases of rapid growth and biodiversity stasis are a reflection of the emergence of bistability (two distinct stable states) in a self-organizing system, which is the Earth’s biota. Transition from one stable state to another is accompanied by a rapid change in the parameters of the system, which may reflect the uneven pace of evolution observed in phylogenetic studies. The reason for the transition from one state to another is the selection of the most adapted individuals, the genetic diversity of which is a result of genome replication errors during self-reproduction. Bistability was found to be characteristic only for ecosystems with predominant sexual reproduction. That is, we explain the characteristics of the Earth’s fossil record during the last 500 million years by the internal laws of Earth’s ecosystem functioning, which appeared at a certain stage of evolution as a result of the emergence of life forms with an increased adaptive diversification associated with sexual dimorphism.

##  Supplemental Information

10.7717/peerj.11130/supp-1 Supplemental Information 1 Derivation of the evolutionary model of a transitive population

10.7717/peerj.11130/supp-2 Supplemental Information 2 Mathematical model of evolutionary self-development of a transitive population Code Model. Software “Mathematica”.

## Additional Information and Declarations

### Data Availability

The following information was supplied regarding data availability: Mathematica code is available as Supplemental File.
