# Supplementary material for: Evolution and extinction can occur rapidly: a modeling approach
Source: PeerJ. 2021 Apr 13;9:e11130. doi: 10.7717/peerj.11130 (PMC8051336; doi:10.7717/peerj.11130)
Supplement: Supplemental Information 1 [file peerj-09-11130-s001.docx]

PUNCTUATED EQUILIBRIUM EVOLUTION: MATHEMATICAL MODEL

Vitaly A. Likhoshvai, Tamara M. Khlebodarova

Supplementary Information

**S1. Derivation of the evolutionary model of a transitive population.**

We have the following evolutionary model of a transitive population (see the main text of the article):

$$\left\{\begin{matrix} \frac{d}{dt}x=k_{in}+x\left(W-Y\right), \\ \frac{d}{dt}C=p_{C}WC\frac{\partial}{\partial C}\left(\frac{d}{dt}F\right), \\ \frac{d}{dt}D=p_{D}WD\frac{\partial}{\partial D}\left(\frac{d}{dt}F\right), \end{matrix}\right.$$

where *x*(*t*) is the density of transitive individuals and W and Y are the laws of self-production and mortality, respectively, which are determined based on the following equations:

$$W=\left(k_{aSx}+k_{Sx}x\right)(1-\frac{x}{C}),$$

$$Y=DH+k_{d}$$

where C and D are the maximum possible biota density and the rate of degradation of transitive individuals, determined by the internal laws of ecosystem functioning, respectively. Since $\frac{d}{dt}F=W_{x}x_{t}+W_{C}C_{t}$, partial derivatives $\frac{d}{dt}F$with respect to the parameters C and D have the following form:

$$\begin{matrix} \frac{\partial}{\partial C}\left(\frac{d}{dt}F\right)=\frac{\partial}{\partial C}\left[W_{x}x_{t}+W_{C}C_{t}\right], \\ \frac{\partial}{\partial D}\left(\frac{d}{dt}F\right)=\frac{\partial}{\partial D}\left[W_{x}x_{t}+W_{C}C_{t}\right], \end{matrix}$$

where

$$\begin{matrix} \frac{d}{dx}W=W_{x}=k_{Sx}-\frac{k_{aSx}+2k_{Sx}x}{C}, \\ \frac{d}{dC}W=W_{C}=\left(k_{aSx}+k_{Sx}x\right)\frac{x}{C^{2}},\frac{\partial}{\partial C}\left(\frac{d}{dC}W\right)=W_{CC}=-2\left(k_{aSx}+k_{Sx}x\right)\frac{x}{C^{3}}, \\ \frac{\partial}{\partial C}\left(\frac{d}{dx}W\right)=W_{xC}=\left(k_{aSx}+2k_{Sx}x\right)\frac{1}{C^{2}}, \\ H=\frac{k_{d}*x*e^{\frac{x}{K_{E}}-1}}{K_{D}+x*e^{\frac{x}{K_{E}}-1}},\frac{d}{dx}H=H_{x}=k_{d}*K_{D}\frac{\left[\left(1+\frac{x}{K_{E}}\right)*e^{\frac{x}{K_{E}}-1}\right]}{\left(K_{D}+x*e^{\frac{x}{K_{E}}-1}\right)^{2}} \end{matrix}$$

Thus, time derivative of the adaptability functional $\frac{d}{dt}F$equals:

$$W_{x}x_{t}+W_{C}C_{t}=W_{x}x_{t}+p_{C}W_{C}WC\frac{\partial}{\partial C}\left[W_{x}x_{t}+W_{C}C_{t}\right]$$

Define as follows:

$$Z=W_{x}x_{t}+W_{C}C_{t}=W_{x}x_{t}+p_{C}W_{C}WCZ_{C}$$

As a result, we obtain an ordinary differential equation:

$$Z=\left[\begin{matrix} \left(k_{Sx}-\frac{k_{aSx}+2k_{Sx}x}{C}\right)\left(k_{in}+x\left(\left(k_{aSx}+k_{Sx}x\right)(1-\frac{x}{C})-Y\right)\right)+ \\ p_{C}x\left(k_{aSx}+k_{Sx}x\right)^{2}(1-\frac{x}{C})\frac{1}{C}Z_{C} \end{matrix}\right]$$

Let us find a particular solution to this equation in the form of a series

$$Z=\sum_{i=0}^{\infty} \left(\frac{z_{i}}{C^{i}}\right),\frac{1}{C}Z_{C}=-\sum_{i=3}^{\infty} \left(i-2\right)\frac{z_{i-2}}{C^{i}}.$$

Derive *Z*.

$$\begin{matrix} \sum_{i=0}^{\infty} \left(\frac{z_{i}}{C^{i}}\right)=\left(k_{Sx}-\frac{k_{aSx}+2k_{Sx}x}{C}\right)\left(k_{in}+x\left(\left(k_{aSx}+k_{Sx}x\right)(1-\frac{x}{C})-Y\right)\right)-p_{C}x\left(k_{aSx}+k_{Sx}x\right)^{2}(\frac{1}{C^{2}}-\frac{x}{C^{3}})\sum_{i=1}^{\infty} i\frac{z_{i}}{C^{i}}, \\ \frac{1}{C^{0}}z_{0}=\left(k_{Sx}\right)\left(k_{in}+x\left(\left(k_{aSx}+k_{Sx}x\right)-Y\right)\right), \\ \frac{1}{C^{1}}z_{1}=\left(-\frac{k_{aSx}+2k_{Sx}x}{C}\right)\left(k_{in}+x\left(\left(k_{aSx}+k_{Sx}x\right)-Y\right)\right)+\left(k_{Sx}\right)\left(x\left(\left(k_{aSx}+k_{Sx}x\right)(-\frac{x}{C})\right)\right)= \\ =-\left[\left(\frac{k_{aSx}+2k_{Sx}x}{C}\right)\left(k_{in}-Yx\right)+x\left(k_{aSx}+k_{Sx}x\right)\left(\frac{k_{aSx}+3k_{Sx}x}{C}\right)\right], \\ \frac{1}{C^{2}}z_{2}=\left(-\frac{k_{aSx}+2k_{Sx}x}{C}\right)\left(x\left(\left(k_{aSx}+k_{Sx}x\right)(-\frac{x}{C})\right)\right)=x^{2}\left(k_{aSx}+2k_{Sx}x\right)\left(k_{aSx}+k_{Sx}x\right)\frac{1}{C^{2}}, \\ \frac{1}{C^{3}}z_{3}=-p_{C}x\left(k_{aSx}+k_{Sx}x\right)^{2}\sum_{i=3}^{3} \left(i-2\right)\frac{z_{i-2}}{C^{i}}=-p_{C}x\left(k_{aSx}+k_{Sx}x\right)^{2}z_{1}, \\ \frac{1}{C^{i}}z_{i}=p_{C}x\left(k_{aSx}+k_{Sx}x\right)^{2}\left[\left(i-3\right)xz_{i-3}-\left(i-2\right)z_{i-2}\right]\frac{1}{C^{i}},i=4,... \end{matrix}$$

As a result, we received a particular solution in the form of a series.

$$\begin{matrix} Z=\sum_{i=0}^{\infty} \left(\frac{z_{i}}{C^{i}}\right),CZ_{C}=-\sum_{i=1}^{\infty} i\left(\frac{z_{i}}{C^{i}}\right),DZ_{D}=DH\sum_{i=0}^{\infty} \left(\frac{w_{i}}{C^{i}}\right), \\ w_{i}=\frac{1}{H}\frac{\partial z_{i}}{\partial D}, \\ w_{0}=-k_{Sx}, \\ w_{1}=x\left(k_{aSx}+2k_{Sx}x\right), \\ w_{2}=0, \\ w_{3}=-p_{C}x^{2}\left(k_{aSx}+k_{Sx}x\right)^{2}\left(k_{aSx}+2k_{Sx}x\right), \\ w_{i}=p_{C}x\left(k_{aSx}+k_{Sx}x\right)^{2}\left[\left(i-3\right)xw_{i-3}-\left(i-2\right)w_{i-2}\right],i=4,... \end{matrix}$$

General system has the form

$$\begin{matrix} \left\{\begin{matrix} x_{t}=k_{in}+x\left(W-Y\right) \\ C_{t}=-p_{C}W\sum_{i=1}^{\infty} i\frac{z_{i}}{C^{i}} \\ D_{t}=p_{D}WDH\sum_{i=0}^{\infty} \frac{w_{i}}{C^{i}} \end{matrix}\right. \\ F=W,Y=DH+k_{d}, \\ W=\left(k_{aSx}+k_{Sx}x\right)(1-\frac{x}{C}),\frac{d}{dx}W=W_{x}=k_{Sx}-\frac{k_{aSx}+2k_{Sx}x}{C}, \\ \frac{d}{dC}W=W_{C}=\left(k_{aSx}+k_{Sx}x\right)\frac{x}{C^{2}},\frac{d}{dC}\left(\frac{d}{dC}W\right)=W_{CC}=-2\left(k_{aSx}+k_{Sx}x\right)\frac{x}{C^{3}}, \\ \frac{d}{dC}\left(\frac{d}{dx}W\right)=W_{xC}=\left(k_{aSx}+2k_{Sx}x\right)\frac{1}{C^{2}}, \\ H=\frac{k_{d}*x*e^{\frac{x}{K_{E}}-1}}{K_{D}+x*e^{\frac{x}{K_{E}}-1}},\frac{d}{dx}H=H_{x}=k_{d}*K_{D}\frac{\left[\left(1+\frac{x}{K_{E}}\right)*e^{\frac{x}{K_{E}}-1}\right]}{\left(K_{D}+x*e^{\frac{x}{K_{E}}-1}\right)^{2}}, \\ z_{0}=\left(k_{Sx}\right)\left(k_{in}+x\left(\left(k_{aSx}+k_{Sx}x\right)-Y\right)\right),z_{1}=-\left[\left(\frac{k_{aSx}+2k_{Sx}x}{C}\right)\left(k_{in}-Yx\right)+x\left(k_{aSx}+k_{Sx}x\right)\left(\frac{k_{aSx}+3k_{Sx}x}{C}\right)\right], \\ z_{2}=x^{2}\left(k_{aSx}+2k_{Sx}x\right)\left(k_{aSx}+k_{Sx}x\right), \\ z_{3}=-p_{C}x\left(k_{aSx}+k_{Sx}x\right)^{2}z_{1}, \\ z_{i}=p_{C}x\left(k_{aSx}+k_{Sx}x\right)^{2}\left[\left(i-3\right)xz_{i-3}-\left(i-2\right)z_{i-2}\right],,i=4,... \\ w_{0}=-k_{Sx}x,w_{1}=\left(k_{aSx}+2k_{Sx}x\right)x,w_{2}=0,w_{3}=-p_{C}x^{2}\left(k_{aSx}+k_{Sx}x\right)^{2}\left(k_{aSx}+2k_{Sx}x\right), \\ w_{i}=p_{C}x\left(k_{aSx}+k_{Sx}x\right)^{2}\left[\left(i-3\right)xw_{i-3}-\left(i-2\right)w_{i-2}\right],i=4,... \end{matrix}$$

A more convenient form of the system describing the evolution of a transitive population:$\begin{matrix} \left\{\begin{matrix} x_{t}=k_{in}+x\left(W-Y\right) \\ C_{t}=-p_{C}W\sum_{i=1}^{\infty} i\frac{u_{i}+Yw_{i}}{C^{i}} \\ D_{t}=p_{D}WDH\sum_{i=0}^{\infty} \frac{w_{i}}{C^{i}} \end{matrix}\right. \\ F=W,Y=DH+k_{d}, \\ W=\left(k_{aSx}+k_{Sx}x\right)(1-\frac{x}{C}),\frac{d}{dx}W=W_{x}=k_{Sx}-\frac{k_{aSx}+2k_{Sx}x}{C}, \\ \frac{d}{dC}W=W_{C}=\left(k_{aSx}+k_{Sx}x\right)\frac{x}{C^{2}},\frac{d}{dC}\left(\frac{d}{dC}W\right)=W_{CC}=-2\left(k_{aSx}+k_{Sx}x\right)\frac{x}{C^{3}}, \\ \frac{d}{dC}\left(\frac{d}{dx}W\right)=W_{xC}=\left(k_{aSx}+2k_{Sx}x\right)\frac{1}{C^{2}}, \\ H=\frac{k_{d}*x*e^{\frac{x}{K_{E}}-1}}{K_{D}+x*e^{\frac{x}{K_{E}}-1}},\frac{d}{dx}H=H_{x}=k_{d}*K_{D}\frac{\left[\left(1+\frac{x}{K_{E}}\right)*e^{\frac{x}{K_{E}}-1}\right]}{\left(K_{D}+x*e^{\frac{x}{K_{E}}-1}\right)^{2}}, \\ u_{0}=\left(k_{Sx}\right)\left(k_{in}+x\left(\left(k_{aSx}+k_{Sx}x\right)\right)\right),u_{1}=-\left[k_{in}\left(k_{aSx}+2k_{Sx}x\right)+x\left(k_{aSx}+k_{Sx}x\right)\left(k_{aSx}+3k_{Sx}x\right)\right], \\ u_{2}=x^{2}\left(k_{aSx}+2k_{Sx}x\right)\left(k_{aSx}+k_{Sx}x\right), \\ u_{3}=-p_{C}x\left(k_{aSx}+k_{Sx}x\right)^{2}u_{1}, \\ u_{i}=p_{C}x\left(k_{aSx}+k_{Sx}x\right)^{2}\left[\left(i-3\right)xu_{i-3}-\left(i-2\right)u_{i-2}\right],,i=4,... \\ w_{0}=-k_{Sx}x,w_{1}=\left(k_{aSx}+2k_{Sx}x\right)x,w_{2}=0,w_{3}=-p_{C}x\left(k_{aSx}+k_{Sx}x\right)^{2}w_{1}, \\ w_{i}=p_{C}x\left(k_{aSx}+k_{Sx}x\right)^{2}\left[\left(i-3\right)xw_{i-3}-\left(i-2\right)w_{i-2}\right],i=4,... \end{matrix}$
